# A review of rural and remote health service indexes: are they relevant for the development of an Australian rural birth index?

**DOI:** 10.1186/s12913-014-0548-7

**Published:** 2014-12-10

**Authors:** Jennifer Pilcher, Sue Kruske, Lesley Barclay

**Affiliations:** University Centre for Rural Health, University of Sydney, Uralba st, Lismore, NSW Australia; University Centre for Mothers and Babies, University of Queensland, St Lucia, Brisbane, Australia

**Keywords:** Indexes, Maternity services, Rural and remote, National maternity services plan

## Abstract

**Background:**

Policy informs the planning and delivery of rural and remote maternity services and influences the perinatal outcomes of the 30 per cent of Australian women and their babies who live outside the major cities. Currently however, there are no planning tools that identify the optimal level of birthing services for rural and remote communities in Australia. To address this, the Australian government has prioritised the development of a rigorous methodology in the Australian National Maternity Services Plan to inform the planning of rural and remote maternity services.

**Methods:**

A review of the literature was undertaken to identify planning indexes with component variables as outlined in the Australian National Maternity Services Plan. The indexes were also relevant if they described need associated with a specific type and level of health service in rural and remote areas of high income countries. Only indexes that modelled a range of socioeconomic and or geographical variables, identified access or need for a specific service type in rural and remote communities were included in the review.

**Results:**

Four indexes, two Australian and two Canadian met the inclusion criteria. They used combinations of variables including: geographical placement of services; isolation from services and socioeconomic vulnerability to identify access to a type and level of health service in rural and remote areas within 60 minutes. Where geographic isolation reduces access to services for high needs populations, additional measures of disadvantage including indigeneity could strengthen vulnerability scores.

**Conclusion:**

Current planning indexes are applicable for the development of an Australian rural birthing index. The variables in each of the indexes were relevant, however use of flexible sized catchments to accurately account for population births and weighting for extreme geographic isolation needs to be considered. Additionally, socioeconomic variables are required that will reflect need for services particularly for isolated high needs populations. These variables could be used with Australian data and appropriate cut-off points to confirm applicability for maternity services. All of the indexes used similar types of variables and are relevant for the development of an Australian Rural Birth Index.

## Background

Approximately 30 per cent of Australian mothers live outside a major city where maternity service provision is limited and birthing services are not always provided locally [1]. Many of these women travel long distances for antenatal care and need to relocate at 36 weeks gestation, to await the birth of their baby [2]. Travelling to access maternity services is a burden financially and socially with a strong correlation between rural and remote residence, travel time and poor perinatal outcomes [3-9]. Consequently those who have the highest health needs often have the least access to services, and is referred to as the ‘inverse care law’ [10,11]. Despite this, there are currently no planning tools that identify the optimal level maternity services in rural and remote communities in Australia.

While health service planners currently combine information on current and projected health needs of a population [12] there is no methodology to identify the level of service required. Once planning is undertaken rural and remote service provision is often negatively influenced by workforce availability or political imperatives [13]. This often results in inconsistent and inequitable planning decisions and limited service provision which impacts most on those who are vulnerable, particularly rural and remote women and children [14,15].

To address this, the Australian Governments have committed to implementing priority actions through the National Maternity Services Plan (NMSP), including the provision of primary maternity services in rural and remote communities [16]. A specific priority action is the “development of a rigorous planning methodology to assist in woman-centred maternity service planning” [16]. The NMSP also lists factors to be considered in service planning design and implementation of maternity services including:

● Birth rates within communities

● Geographic factors such as remoteness

● Socioeconomic factors including community levels of disadvantage

● Links to medical specialists

● Resourcing and service capability

An index using the range of variables listed above could be the basis for developing a rigorous methodology for planning. An index is a mathematical construct that integrates a group of variables, relevant to the purpose for which it is created [17,18]. For an index to inform planning of health services, the variables used need to demonstrate a relationship to the service being described such as those outlined in the NMSP. It should also be simple to use and constructed with variables that use current easily available data [19]. The measures for each variable need to be weighted according to the health needs in communities. The final scores in an index are then calibrated with cut-offs between scores commensurate with levels of service appropriate for rural and remote communities [17].

A review of the literature was undertaken to identify indexes associated with planning rural and remote services, using variables listed in the NMSP. Components of each index were then assessed to determine their relevance in the development of an Australian rural birthing index.

## Methods

**Figure 1:**
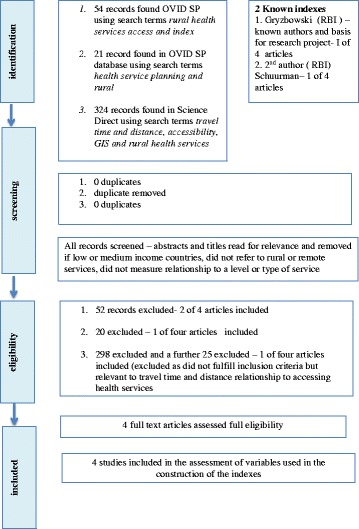
**Literature search flow diagram for rural and remote indexes.**

A search of the literature was undertaken in two steps. The first step was a search of the electronic databases OVID SP (Medline, Psychinfo) Embase, Informit and Science Direct to identify any indexes, service planning or decision making tools (Figure 1). The searches were limited to English language articles and articles from the year 1990 onwards. Search terms used include: decision- making tools; modelling; indexes; prioritisation tools; rural and maternity health service planning; policy development; accessibility and access; rural and remote health and maternity services.

## Literature review

### All Search terms used 

Stage 1 index search excluding index author searches

Rural

Rural population

Rural and remote health

Rural and remote health services

Rural health services, access and index

Rural health services and models/modelling

Rural health services/Rural hospital, levels of service

Health service planning,

Accessibility, access

Health service accessibility

Accessibility Regional Health Planning/

Need, rural populations

rural, remote health service planning

Health service delivery

Maternity services

Rural and remote maternity services

Pregnancy/or Maternal Health Services/or Australia/or Midwifery/

women's health services/or "

health care quality, access, and evaluation"

geographical, geography access and health.

Access, Transport- rural, Geographical information systems (GIS) and accessibility of health services

Australia, UK, Canada, Scotland, New Zealand

Decision Making, Index, modelling, models, tools, decision making tools and frameworks

Stage 2 variable search

Rural health services

Maternity health services

Hospital catchments, health service catchments

Emergency transfers and maternity Transportation of Patients" Emergency Medical Services, Patient Transfer, pregnancy, perinatal care, Inter-hospital transfers and outcomes, Aeromedical Retrievals and maternity services, RFDS, rural and remote transfers, delivery of health care

Quality of Health Care", Obstetric Labor Complications/Delivery, Obstetric/Obstetrics/Pregnancy Complications/Pregnancy/Maternal Mortality/Maternal Health Services/Health Services Accessibility childbirth or pregnancy complications or rural areas or prematurity or mothers or health

Infant, Newborn/ or Pregnancy/or Prenatal Care/or Maternal Health Services/or Mothers/or Midwifery/or Obstetrics/

Distance and time travelled and access

accessibility, GIS and rural health services

Socioeconomic status, need

Isolation, indigenous, travel and maternal outcome, remote area health and outcome, remote area health and perinatal outcomes,

maternal health services/or perinatal care/or preconception care/or prenatal care/or health services, indigenous/or "health care quality, access, and evaluation"

Limits applied

● Humans only

● English Language

● 1990- current

The inclusion criteria included indexes that:

● Inform access to a level or type of service in rural and remote communities in high income countries

● Were congruent with the factors listed in the NMSP

Once indexes were identified, a second search was undertaken to explore’ the variables and related measures used in the construction of the indexes. OVID SP- Medline, Science Direct and GEOBASE databases were searched using the terms accessibility of services; distance and travel time; catchment areas and socioeconomic vulnerability. Where an article was considered highly relevant such as index or specific variable paper, the search was broadened to include all authors, reference lists and citations of the relevant articles.

Articles were excluded when studies were based in low and or medium income countries, did not associate with rural and remote health services or a level or type of service or were clinically rather than service focused (Figure 1). Indexes were also excluded if they were used for funding requirements, measured utilisation of services and only used a single variable such as socioeconomic deprivation [18-21].

## Results

Four indexes were identified that fulfilled the inclusion criteria. Two of these were Australian [22,23] and two Canadian [17,24]. Each index was constructed using variables that linked access to rural and remote health services through integrated geographic and or socioeconomic variables (Table 1).

**Table 1:** **Comparison of variables used in each of the four identified indexes**

**Index**	**1. Rural birth index [** 17 **]**	**2. Trauma model [** 24 **]**	**3. Index of rural access [** 23 **]**	**4. Cardiac aria index [** 22 **]**
**Service**	Maternity	Trauma	GP* services	Cardiac emergency & cardiac rehabilitation
**Level or capability of service**	levels of maternity service dependent on level of staff and procedural care available	Level of trauma service- I,II,III- complexity of trauma care	Primary care services	**AIHW Hospital types large -small including community level services
**Population**	Rural British Columbia, Canada	Rural British Columbia, Canada	Rural Victoria, Australia	Total Australian- rural and urban pop locations (20,387)
**Catchment**	60 minute to a ***CS service	60 minute to a trauma service	Ration of *GPs to population in a 60 minute catchment	Population access to a service within 60 minutes for an emergency cardiac response
**Index Specific**	Birth numbers within the 60 minute catchment of a service with ***CS capability	Risk of trauma, ^SES and access to trauma service	Ratio of *GP services to population in a 60 minute catchment	Access to emerg care in a cardiac event and for cardiac rehabilitation
**Isolation**	Seven categories of time <30 min- >4 hrs to a service with CS capability	travel time to trauma centre- highest quintile of need assigned to least serviced communities (metro excluded)	60 min catchments- ‘distance decay’ after 10 minutes	Decreasing levels of services as remoteness increases- 8 levels hospital
**Vulnerability**	1 data set ^SES advantage-disadvantage	^^VANDIX ^^^SEFI	6 SES measures that impact health outcomes including: Indigenous & #CALD included	N/A
		^SES vulnerability to trauma		
**Emergency treatment**	one hour an important threshold for emergency care	One hour critical time to treatment for trauma	N/A	One hour critical time to treatment in a cardiac event

*GP– general practitioner, **AIHW- Australian Institute of Health and Welfare, ***CS- caesarean section.

^SES Socioeconomic status, ^^VANDIX- Vancouver Area Neighbourhood Deprivation Index, ^^^SEFI- Socioeconomic Factor Index.

#CALD- culturally and linguistically different.

Only one of the four indexes was associated with maternity services, however, all four of the indexes used variations of relevant and similar variables. All four indexes were composed of variables using measures of travel time to, or isolation from a service, catchment size, service specific identifiers and three of the four used measures of socioeconomic vulnerability [17,23,24] Table 1. While ‘emergency treatment’ was not a defined variable per se, it was explicitly related to access for the cardiac and trauma indexes [22,24] and the maternity index stated that one hour was a threshold for emergency care [17] (Table 1).

The index specific to the planning of maternity services in rural areas was the Canadian Rural Birth Index [17]. Its variables include: population birth numbers within a sixty minute catchment; an isolation factor measuring time to caesarean section in half hour intervals up to four hours; and, a vulnerability factor measuring socioeconomic status. Levels of maternity service identified in the Rural Birth Index are descriptive but comparable to the definitions of levels of service in the Australian National Maternity Services Capability Framework (NMSCF) [25]. The Rural Birth Index ‘Antenatal and postnatal services only’, equate to NMSCF Level 1 services, ‘birthing without operative services’ equates to NMSCF Level 2 and ‘birthing services with caesarean section’ equate to NMSCF Level 3 services [17,25]. When tested the Rural Birth Index demonstrated an 80 per cent accuracy rate for identifying the level of maternity service required for rural communities in British Columbia [17].

The Trauma Model is also a Canadian tool that identified communities with ‘good’ access to trauma services, and those in need of trauma services, using the American College of Surgeons level of trauma service definitions [24]. The Trauma Model incorporates measures of isolation and time to a trauma service, social vulnerability, and a risk of trauma measure as variables [24]. The Rural Birth Index and Trauma Model share common authors and are both associated with health service access in British Columbia, Canada. The Trauma model was validated using trauma services utilisation data and found that socioeconomic vulnerability measures strengthen the model’s use of geographic and travel time measures [24].

The Index of Rural Access is Australian and uses Victoria as the population base to map access General Practitioner (GP) services [23]. It combines four key elements of access including availability of services, proximity to services, health needs and mobility. This is achieved through the identification of catchments and related travel time to GP services for rural populations. Due to higher population density in many parts of rural Victoria than other parts of rural Australia, there is more likely to be increasing choice and access to services for people depending on the number of GPs in a one hour catchment.

The Cardiac Aria Index used geographical information systems (GIS) to map 20,387 urban, rural and remote population locations against the availability of local health services including non-cardiac specific ambulances and pharmacies [22]. The Cardiac Aria Index used Australian Institute of Health and Welfare (AIHW) hospital definitions associated with the level of activity in each type of hospital [26]. The AIHW definitions of hospital can be linked to a level of cardiac service defined in one of the various capability frameworks in use in Australia [27-29]. The Cardiac Aria index differs from the three other identified indexes as socioeconomic variables were not used.

Each of the variables used in the indexes will now be considered in more detail to assess their suitability for inclusion in the development of an index for use in planning maternity services in rural and remote Australia.

### Catchment

Catchments are designated geographic areas with a relationship to a service or subject of interest [14,30], for example, the population of births within one hour travel time of a health facility Rural Birth Index. The size of the catchment can vary, dependent on the level and type of service, population size and density and the reasons for determining the catchment. Despite the geographic relationship between the location of a service and the community, it is the populations’ perception of service accessibility that impacts on use of the service [4,31-33]. As a result defined hospital or service catchments do not guarantee that the population will use that service, only that the service is available to a recognised population [23,30].

An index however, requires that a consistent measure of population size is used to identify what number of the population can reasonably be expected to use the service within a given travel time [14,34]. Canada has prescribed in legislation and policy, maximum acceptable travel times to categories of service [34] while Australia has not. Despite differences in legislation between Canada and Australia, accessibility using travel time of one hour has become an accepted measure of access particularly in an emergency [17,22,24,34].

### Isolation

Services accessible within a one hour travel time, is considered good access [14,24,30]. Increasing distance outside of this one hour travel time norm is considered to be a measure of isolation. The Rural Birth Index measured isolation by travel time to a service providing caesarean section [17]. The isolation factor used seven categories of time weighted in a stepwise fashion from less than 30 min to greater than four hours [17] with higher scores for longer time categories to a service. The Trauma Model used GIS to calculate travel time from both rural and urban population areas to the nearest trauma centre (Level 1, 2 or 3) [24]. It was the most isolated and socio economically disadvantage communities that were at highest risk of trauma that were found to be in greatest need of trauma services. The combination of geographic isolation and socioeconomic disadvantage amplified the effect of need for a trauma service. In the Index of Rural Access, ‘isolation’ measured the lack of mobility or transport to access services, generally associated with socioeconomic disadvantage [23].

### Vulnerability

The relationship of social disadvantage to health outcomes is well established regardless of service type, especially for those living in rural and remote areas and those who identify as indigenous [3,15,35,36]. Socio economic vulnerability was measured in three of the four indexes: the Rural Birth Index, the Trauma Model and the Index of Rural Access. The Cardiac Aria Index was the only index that did not include socioeconomic variables. However it did identify that lack of access to cardiac services was highest for vulnerable populations including indigenous people and those aged over 65 [22]. Each index used different types of census data as either single variables or aggregate groups of data based on identified geographical areas. Use of census data to quantify socioeconomic deprivation is a well-accepted method of identifying populations with poorer health outcomes [19].

The Rural Birth Index used previously validated British Columbia Statistics Socioeconomic Indices (BCSSI). The BCSSI measures the social vulnerability of a local health area ranging from socially disadvantaged to socially advantaged. The Trauma Model used two types of aggregate socioeconomic scores of disadvantage including a measure of trauma risk, correlating the likelihood of increased rates of trauma with socioeconomic status [22]. The Index of Rural Access was also the only index to explicitly include an indigenous variable and identify car ownership as a proxy for mobility [23]. Decisions to access services are influenced by socioeconomic vulnerability and its influence on mobility through access to transport or car ownership [23].

## Discussion

The benefits of using an index include more equitable and consistent planning decisions and identification of the appropriate level of service to meet the needs of rural and remote communities [17]. An index developed specifically to identify the level of service required for rural and remote communities, could provide the evidence for reinstating or providing new maternity services particularly in high-need communities. However, the use of an index is warranted only if the selection of variables are weighted appropriately, can measure the population need for a service, identify the level of service required, use cut-offs that are appropriate for Australian maternity services and be easy to use [17,19,24].

The relevant variables identified in this review are prerequisites for categorising need and include: population birth numbers; various socioeconomic vulnerability measures that encompass service specific issues including indigeniety; isolation or distance from a service; access to emergency care and catchment size. These variables are consistent with those listed in the NMSP, but also include additional variables that would be suitable for inclusion in the development of an Australian rural birthing index. The importance of each variable in the Australian context is now considered.

### Catchment and population birth numbers

The geographical size of a catchment is a key variable to be included in any rural birthing index and informs the number of population births. Population births are the main predictor of need for maternity services and an indicator of the level of service required [17]. Therefore the catchment needs to accurately reflect the population who might use a services. In rural and remote areas of Australia a catchment is often congruent with a health administrative or geographical population area, rather than one hours travel time from a service. There are no legislated travel times related to accessibility in Australia as there are in Canada, [14] and often only one hospital or health service to provide services across a wide geographic area.

An Australian Rural Birth Index would need to be flexible enough to adjust the travel times and associated catchment sizes where appropriate. The catchment size will depend on the geographical area from which patients flow to access services, and the type of service being planned. Therefore limiting catchment size to one hour travel time would not always accurately reflect the population births in a geographic area. This in turn would impact on the level of service indicated in some areas of remote Australia.

Increasing the catchment size to suit Australian conditions, however, needs to be balanced against the fact that time to treatment can be a critical outcome measure as seen in three of the four indexes examined [17,22,24]. There is also evidence for poorer outcomes and higher intervention rates for women who must travel greater than 60 minutes for maternity services, even when caesarean section is not required [3,37]. Travel time to services is not only a critical factor for access to treatment but increasing travel time also impacts on the decisions people make to access health services and is identified as ‘distance decay’ in the Index of Rural Access [23]. While one hour travel time has become an accepted measure of access to services, an Australian rural birth index would need to identify a catchment area that best captures the reality of patient flows within a geographic area of a maternity service.

### Isolation

Isolation refers to the increasing distance patients must travel to access services beyond one hour. The reality of travel in Australian rural and remote areas is that travel time to services often takes many hours, utilising multiple forms of transport. While the Rural Birth Index used time categories up to four hours, this may be due to the requirements of Canadian legislation. The Canadian Ministry of Health has legislated that categories of health services be accessible to 98 per cent of the population within one hour for emergency services, two hours for acute inpatient services and four hours for speciality services [14,34]. Australia does not have legislation that directs categories of health services to be provided within particular timeframes. However, isolation scores in an Australian index could be weighted sufficiently to address travel time to services greater than four hours. Weighting isolation scores appropriately would assist in identifying those remote communities where population birth numbers and need for services are sufficient enough to warrant birthing services.

Paradoxically the negative impact of relatively short travel times, from 20 to 45 minutes, on perinatal outcomes reinforces that maternity services, as identified in the first principal of the NMSP, should be delivered ‘close to home’ [16]. The impact of travel time and distance on perinatal outcomes makes it imperative that the appropriate level of birthing service with a skilled workforce is available locally [3]. In Canada, women who had to travel one to two hours from a services were more likely to have an unplanned out of hospital birth and neonatal mortality was three time more likely if women had to travel four hours or more [4,37]. The Independent Reconfiguration Panel for maternity services in the UK decided not to close a facility and merge maternity services, because of evidence indicating that long journey times of up to 90 minutes in a rural area were considered unsafe [38]. In the Netherlands there was an increase in perinatal mortality and adverse outcomes for car journeys of 20 minutes or more to a hospital [39] and in a UK population study on stillbirth risk and distance travelled, risk increased when journeys were over 25 kilometres [40].

Although the time to emergency treatment is not a specific variable in any of the indexes identified, it is implicit in the relationship of distance and time to critical treatment requirements and access to services [17,22,24,41,42]. The Rural Birth Index uses the threshold for appropriate access to emergency care of one hour and measured access to a caesarean section as a key variable [17]. As with the Rural Birth Index, an Australian index would need to provide higher scores with increasing distance from services [17] not only because of need for routine maternity care but to provide local access to care in an emergency. It is also relevant to allocate higher scores where isolation from services and socioeconomic disadvantage compound the inability to access services and the effect on perinatal outcomes.

### Vulnerability

Social disadvantage impacts on the ability to access services in all environments. However, overcoming barriers to access, both routinely and in an emergency is worsened by increasing remoteness or isolation. [19,24,43]. Australian perinatal outcomes are particularly vulnerable to socioeconomic disadvantage [44], especially for rural and remote women who identify as Indigenous [2,15,36]. Indigenous mothers tend to be younger than non-Indigenous mothers and experience worse infant mortality rates [35]. ‘Birthing on Country’ is also recognized as being of significant cultural importance for Aboriginal women, where considerable distress is caused by the relocation of Aboriginal women to regional centres at 36–38 weeks gestation to await labour and birth [15,45,46].

Variables used in an Australian rural birthing index would need to reflect the characteristics of socioeconomic vulnerability and health needs of rural and remote populations. An Australian index could use aggregated census data as it is accessible is updated regularly, is place dependent and describes the vulnerability of the population. Census data is collected at five-year intervals in Australia is aggregated into socioeconomic indexes for areas (SEIFA) scores by the Australian Bureau of Statistics and is publically available for analysis [47] as is Australian perinatal data.

Using one SEIFA score of disadvantage can identify the level of need for services in a community such as in the Rural Birth Index [17]. However, increasing the number of socioeconomic variables strengthens the relationship of disadvantage to need and the socioeconomic effect on geographic variables in the index such as in the Trauma Model [19,24]. Additional single socioeconomic variables applicable to a specific community could strengthen the weighting for vulnerability, as aggregate census data gives only a broad assessment of area SES [18,19,24]. Adding variables such as car ownership and an indigenous weighting in an index as seen in the Index of Rural Access could increase the score for high needs communities and more adequately indicate the level of service required. Cars are often the only method of travel in rural and remote Australia, where public transport is limited. Car ownership is related to socioeconomic status and impacts on people’s ability to access services including a woman’s ability to access appropriate care at all stages of the maternity service continuum [5,23,48].

## Conclusion

This paper aimed to identify indexes associated with planning rural and remote services using variables listed in the NMSP. The variables identified in each of the indexes were relevant to the construction of Australian index but require modification depending on the population under consideration. Catchment size would need to accurately reflect the population births and the patient flows in a geographic area with appropriate scores for remoteness and isolation. Aggregate variables of socioeconomic disadvantage using SEIFA scores and possible use of individual variables such as car ownership, age and education level could provide additional weighting particularly for high needs remote communities. In addition a variable for communities with a high proportion of indigenous population could be added. These considerations would strengthen vulnerability scores, particularly where geographic isolation reduces access to services for high needs populations. The development of an Australian rural birthing index would use specific Australian data, with appropriate cut-off points tested to confirm its applicability. All of the indexes used similar types of variables and are relevant for the development of an Australian rural birth index.

## Abbreviations

AIHW: Australian Institute of Health and Welfare
GIS: Geographical information systems
GP: General practitioner
NMSP: National Maternity Services Plan
SEIFA: Socioeconomic indexes for areas
